# Roles of Non-coding RNAs and Angiogenesis in Glioblastoma

**DOI:** 10.3389/fcell.2021.716462

**Published:** 2021-09-27

**Authors:** Ebrahim Balandeh, Kimia Mohammadshafie, Yaser Mahmoudi, Mohammad Hossein Pourhanifeh, Ali Rajabi, Zahra Razaghi Bahabadi, Amir Hossein Mohammadi, Neda Rahimian, Michael R. Hamblin, Hamed Mirzaei

**Affiliations:** ^1^Department of Clinical Psychology, School of Medicine, Kashan University of Medical Sciences, Kashan, Iran; ^2^Department of Biology, Faculty of Science, Golestan University, Gorgan, Iran; ^3^Department of Anatomical Sciences, Yasuj University of Medical Sciences, Yasuj, Iran; ^4^Razi Drug Research Center, Iran University of Medical Sciences, Tehran, Iran; ^5^School of Medicine, Kashan University of Medical Sciences, Kashan, Iran; ^6^Research Center for Biochemistry and Nutrition in Metabolic Diseases, Institute for Basic Sciences, Kashan University of Medical Sciences, Kashan, Iran; ^7^Endocrine Research Center, Institute of Endocrinology and Metabolism, Iran University of Medical Sciences, Tehran, Iran; ^8^Laser Research Centre, Faculty of Health Science, University of Johannesburg, Doornfontein, South Africa

**Keywords:** glioblastoma, non-coding RNAs, angiogenesis, exosomes, microRNAs, long non-coding RNAs

## Abstract

One of the significant hallmarks of cancer is angiogenesis. It has a crucial function in tumor development and metastasis. Thus, angiogenesis has become one of the most exciting targets for drug development in cancer treatment. Here we discuss the regulatory effects on angiogenesis in glioblastoma (GBM) of non-coding RNAs (ncRNAs), including long ncRNA (lncRNA), microRNA (miRNA), and circular RNA (circRNA). These ncRNAs may function in *trans* or *cis* forms and modify gene transcription by various mechanisms, including epigenetics. NcRNAs may also serve as crucial regulators of angiogenesis-inducing molecules. These molecules include, metalloproteinases, cytokines, several growth factors (platelet-derived growth factor, vascular endothelial growth factor, fibroblast growth factor, hypoxia-inducible factor-1, and epidermal growth factor), phosphoinositide 3-kinase, mitogen-activated protein kinase, and transforming growth factor signaling pathways.

## Introduction

Blood vessels function as channels for delivering nutrients and oxygen as well as removal of the metabolic waste products, *via* a capillary network (Hernández-Romero et al., 2019). Angiogenesis has important roles in tissue remodeling and homeostasis in adults (female reproductive cycle and wound healing), in embryonic development, and notably in cancer initiation and progression (Carmeliet and Jain, 2011a; Song et al., 2014). Angiogenesis is dependent on the balance of anti-angiogenic with the pro-angiogenic variables [including thrombospondin (TSP)-1/2, vascular endothelial growth factor (VEGF), and platelet-derived growth factor (PDGF)]. The process of angiogenesis includes other mechanisms of neovascularization, like vasculogenic mimicry, vessel co-option, lymphangiogenesis, vasculogenesis, and intussusceptive angiogenesis (Xu X. et al., 2019).

The role of angiogenesis in tumor development has been studied for the last 50 years. It is now acknowledged that angiogenesis is also critical for the establishment and dissemination of metastatic tumors, as well as the progression of primary tumors (Bielenberg and Zetter, 2015). Angiogenesis enables tumors to grow beyond a specific minimum size and also serves to increase metastasis. Under hypoxic conditions, cancer cells shift this balance toward the increased secretion of pro-angiogenic factors, like VEGF and angiopoietin to facilitate neo-vascularization (Hanahan and Weinberg, 2000; Zheng et al., 2004). The above trend has been named the “angiogenic switch,” leading to the activation of the endothelial cells in the adjacent vessels. Then, the extra-cellular matrix (ECM) is degraded by various proteolytic enzymes to allow the activated endothelial cells to migrate along the chemotactic gradient leading toward a tumor, and which then form vessel-like structures (Carmeliet, 2000). These neovascular structures are fragile and immature, and tend to be very leaky. Once the capillary tubes are formed, the further growth of the endothelial cells is suppressed, and pericytes as well as smooth muscle cells are eventually recruited for blood vessel maturation. Although these new vessels are disorganized, they are still able to supply the growing tumor mass with the required oxygen and nutrients (Folkman, 2002). In spite of numerous data on the pathologic mechanism of angiogenesis, there is still incomplete information about all of the molecular pathways that can trigger the angiogenic switch.

Non-coding RNAs (ncRNAs) have been discovered as novel factors with a wide ability to modulate the expression of many (if not most) genes (Dalmay and Edwards, 2006). These ncRNAs exert their gene modulatory functions at multiple different levels, such as post-translational and post-transcriptional levels. Herein, we highlight the role of ncRNAs, which are correlated to angiogenesis in GBM.

## Angiogenesis and GBM

**FIGURE 1:**
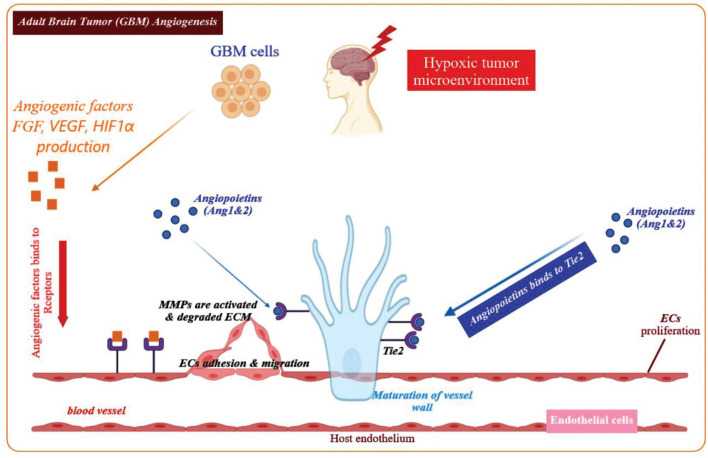
Overview of the angiogenic process in GBM. Angiogenesis is initiated by angiogenic factors released from the GBM cells in the microenvironment of a hypoxic tumor. Key angiogenic factors contributing to the GBM angiogenesis process include, FGF, VEGF, Ang-1, Ang-2, and HIF1α. These factors bind to their respective receptors on the endothelial cells and stimulate migration and proliferation of the endothelial cells. The ECM is gradually degraded and in the next phase, the endothelial cells assemble into a tube or vessel-like structure. It is notable that maturation of the blood vessel walls is accompanied by recruitment of pericytes that cover the endothelial cells from the outer surface, in order to construct a new functional blood vessel.

Physiological angiogenesis is crucial for delivering a sufficient supply of oxygen and nutrients to developing tissues and wound healing (Chung et al., 2010). Angiogenesis involves several steps requiring different elements, including cells (mural and endothelial cells), matrix components and adhesion proteins, proteolytic enzymes, and soluble growth factors as shown in Figure 1 (Carmeliet, 2005). Hypoxia is the major stimulus that activates hypoxia-inducible factor-1α (HIF-1α), which is a transcription factor governing the expression of metabolic proteins, adhesion molecules, matrix components, and growth factors (Fitsialos et al., 2008; Wise et al., 2011). Angiogenesis induction depends on the balance of anti-angiogenic and pro-angiogenic factors. Oxygen deficiency within the cell triggers HIF-1α stabilization, and the secretion of pro-angiogenic growth factors, such as epidermal growth factor (EGF), angiopoietin-1, fibroblast growth factors (FGFs), transforming growth factor-β (TGF-β) as well as VEGF (Rifkin and Moscatelli, 1989; Stacker et al., 2001). Such angiogenic factors bind to their respective receptors, leading to disruption of the existing vessel walls, degradation of the endothelial basement membranes as well as ECM. Upon the degradation of the basement membranes, proteases such as matrix metallo-proteinases (MMPs) will remodel ECM components and generate a new matrix to support stromal cells and enhance the migration and proliferation of endothelial cells, eventually leading to the formation of capillary tube-like structures (Lakka and Rao, 2008). Moreover, the newly formed endothelial tubes become surrounded by a more mature vascular-basement membrane, which is synthesized by mural cells (pericytes and smooth muscle cells) leading to a new stable blood vessel (Figure 1; Hong et al., 2014).

It is well known that angiogenesis is important for tumor progression and growth. Cancerous cells experience hypoxic conditions because of the limited capacity of the blood supply to transport sufficient oxygen. Hypoxia triggers the cancer stem cells to differentiate into mature endothelium that creates novel vessels in the tumor (Carmeliet and Jain, 2011b). Vessels which develop in primary tumors are larger compared to their respective normal counterparts, creating a criss-cross network, with irregular lumen diameters, largely permeable to macromolecules, dilated, and irregularly branched (De Bock et al., 2011). Moreover, the neovascular permeability results in plasma extravasation leading to clotting and localized edema (Mazzone et al., 2009; Nagy et al., 2010). This increased interstitial pressure in the tumor vasculature reduces the influx of leukocytes and lessens the penetration of drugs into the tumor mass (Folkins et al., 2009). Furthermore, due to the lack of perivascular connective tissue barriers as well as the defective basement membrane, cancerous cells may invade into adjacent or distant tissues (Papetti and Herman, 2002). Vessel compression and leakiness leave large tissue volumes in the tumor without blood circulation, and block delivery of nutrients and oxygen leading to necrotic areas occurring within the tumor, particularly in the center (Baish et al., 2011; Stylianopoulos and Jain, 2013). Ischemic conditions lead to stabilization of hypoxia activating HIF-1α, which results in new vessel generation (Semenza, 2001). The defective vasculature leads to resistance to anticancer drugs as well as inhibiting the attack by the host immune system. Considering the contribution of angiogenesis to the growth of tumors, suppression of pro-angiogenic growth factors and targeting the tumor neovasculature can be effective practical approaches for tumor treatment (Ahir et al., 2020).

## MicroRNAs Biogenesis and Functions

**FIGURE 2:**
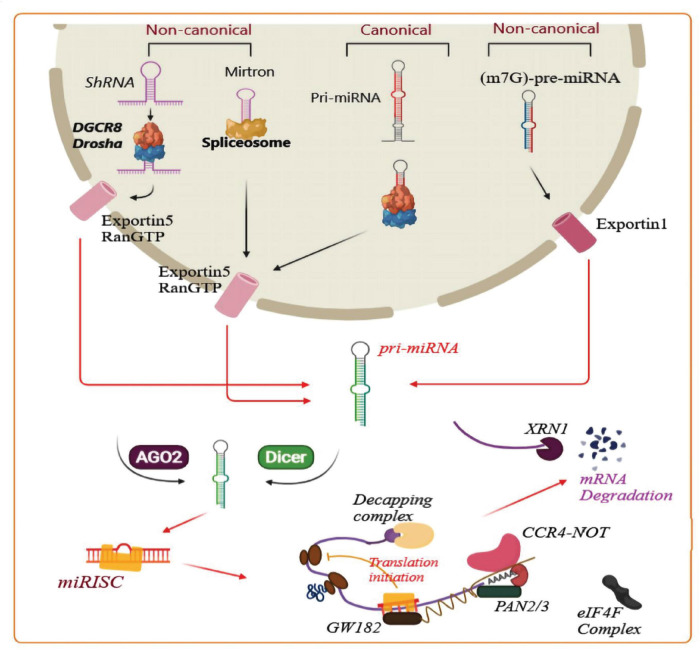
Biogenesis of microRNAs and related mechanisms. A pri-miRNA transcript is the first stage of canonical miRNA biogenesis, which is cleaved by a micro-processor complex (containing Drosha and DGCR8), to create the precursor-miRNA (pre-miRNA). Then, the Exportin5/RanGTP-dependent pathway transports the pre-miRNA to the cytoplasm and processes it to produce a mature miRNA duplex. In the next step, the 5p or 3p strand of the mature miRNA duplex is loaded into a member of the Argonaute (AGO) family of proteins to form a miRNA-induced silencing complex (miRISC). Next, the small hairpin RNA (shRNA) is cleaved in a non-canonical pathway, and Exportin5/RanGTP transports it to the cytoplasm and processes it through an AGO2-dependent, but Dicer-independent cleavage mechanism. 7-Methylguanine capped (m7G)-pre-miRNA and mirtrons depend on Dicer for completing their cytoplasmic maturation, however there are differences in their nucleocytoplasmic shuttling. Exportin5/RanGTP exports mirtrons whereas Exportin1/m7G exports pre-miRNAs. Each pathway results in a functional miRISC complex which can recognize its target mRNA to induce translational suppression *via* interference with the eIF4F complex. Then, the poly(A)-deadenylases PAN2/3 and CCR4-NOT are recruited by the GW182 family protein attached to Argonaute, and PAN2/3 starts deadenylation while this process is completed by the CCR4-NOT complex, and finally the m7G cap on the target mRNA is removed *via* the decapping complex. Finally, the decapped mRNA undergoes 5′-3′ degradation through exoribonuclease XRN1 (modified from the results reported in Hayder et al. (2018) study).

MicroRNAs (miRNA) are a class of non-coding, single-stranded RNA 21–25 nucleotides in length (Nahand et al., 2020). MiRNA biogenesis has been categorized into canonical and non-canonical pathways. Canonical and some non-canonical miRNA biogenesis pathways are shown in Figure 2. MiRNA plays a pivotal role in RNA silencing and post-transcriptional regulation of gene expression, thereby taking part in numerous cellular processes, such as proliferation, apoptosis, cell cycle progression, migration, and differentiation (Lou et al., 2019; Nahand et al., 2020). The dysregulation of miRNAs is linked with multiple human diseases. Many studies have shown that there is a close relationship between miRNAs and human cancer (Tutar, 2014). MiRNAs have also been reported to exert key functions in the onset and progression of GBM (Lou et al., 2019). However, the exact mechanism has not been clearly defined.

## MiRNAs and Angiogenesis in Glioma

Myc-associated zinc finger protein (MAZ) is an ubiquitously expressed transcription factor (Song et al., 1998) which is able to modulate the expression of several genes, including serotonin receptors (Parks and Shenk, 1996), insulin (Kennedy and Rutter, 1992), and c-*myc* (Bossone et al., 1992). Researchers have also detected MAZ-binding sites within the promoter region of several pro-angiogenic genes (Ray et al., 2004; Sohl et al., 2009), like VEGF (Ray et al., 2007).

Smits et al. (2012) reported that the expression level of miR-125b was decreased in the glioblastoma (GBM)-associated endothelial cells along with an increase in the expression of its target (MAZ). In addition, in endothelial cells exposed to VEGF or to GBM-conditioned medium, the level of miR-125b was higher, and the level ofMAZ was correspondingly higher. Also, they found that the increased levels of MAZ could be inhibited by MAZ-specific shRNAs or by over-expression of miR-125b, leading to decreased migration of primary human brain endothelial cells and less tubule formation *in vitro*. Moreover, they found that the expression level of MAZ was increased in brain blood vessels from GBM patients. Overall, in GBM-associated angiogenesis, these findings indicate a functional feed-forward loop, in which VEGF suppresses miR-125b expression, leading to elevated MAZ expression, which in its turn causes transcriptional activation of VEGF. Functionally, the above loop is inhibited by the VEGF receptor inhibitor, vandetanib that points toward the further development of tumor-angiogenesis inhibitors (Smits et al., 2012).

As a full-length tyrosine kinase receptor, Fms-related tyrosine kinase 1 (Flt1) is characterized by its extra-cellular domain, and is expressed on the endothelial cells of blood vessels. Therefore, researchers suggested its involvement in angiogenesis as well as tumor progression (Ferrara et al., 2003). Moreover, the Wnt/β-catenin pathway is one of the conserved signaling pathways with a major role in several cancers like glioma (Wang et al., 2014; Mir et al., 2016). A study by Wang and co-workers also showed that miR-139-5p inhibited the Flt1-induced Wnt/β-catenin pathway in glioma cells (Wang Q. et al., 2018). They showed that miR-139-5p was down-regulated in glioma tissues in comparison to healthy brain tissue, and that forced expression of miR-139-5p led to down-regulation of Flt1 by direct binding of miR-139-5p to the 3′UTR of Flt1. In addition, they confirmed that miR-139-5p targeted Flt1 by carrying out a rescue experiment. They showed that Flt1 stimulated and miR-139-5p inhibited the proliferation of the glioma cells (Wang Q. et al., 2018).

According to the literature, Wnt5a is a typical ligand of the non-canonical Wnt pathway. Among the possible Wnt5a downstream pathways, researchers have shown that Wnt/Ca2^+^ and planar cell polarity (PCP) pathways are implicated in normal cell physiology and cancer progression (Asem et al., 2016). The PCP pathway regulates morphogenetic movement and cell polarity by activating c-Jun N-terminal protein kinase (JNK) (Pu et al., 2009; Asem et al., 2016). Finally, Wnt/Ca2^+^ pathway regulates the motility and adhesion of cells *via* activation of calmodulin-dependent protein kinase II, protein kinase C (PKC), and phospholipase C (Pu et al., 2009; Asem et al., 2016).

Zeng et al. (2018) demonstrated the miR-129-5p contribution to GBM. They found that the expression level of miR-129-5p was down-regulated and there was an inverse correlation between miR-129-5p and Wnt5a in the CGGA glioma patient samples. Restoration of miR-129-5p expression *in vitro* decreased resistance to temozolomide, and inhibited neurosphere formation, angiogenesis, migration, invasion, and proliferation of GBM cells. Moreover, it was shown that miR-129-5p directly targeted Wnt5a leading to a decrease of Wnt5a expression, and inhibited JNK and PKC/ERK/NF-κB pathways. The ability of miR-129-5p to inhibit tumor growth was demonstrated utilizing an *in vivo* xenograft mouse model. This research suggested that the miR-129-5p/Wnt5 axis affects the JNK and PKC/ERK/NF-κB signaling pathways, and has the potential to be used in GBM therapy (Zeng et al., 2018).

The RAS sarcoma (Ras) gene is one of the most commonly activated oncogenes in many human cancers. N-RAS, H-RAS as well as K-RAS are the prototypical members of a family of small G-proteins often activated in human tumors, such as glioma. Researchers have shown that K-RAS was a miR-143 target in colorectal cancer cells. N-RAS is involved in some cancers such as glioma, and contributes to angiogenesis, invasion, migration, survival, and proliferation of the cells (Cantley, 2002). Increased N-RAS gene expression may be a key factor in glioma progression (Tsurushima et al., 1996). Tumorigenesis is promoted by oncogenic N-RAS *via* activating several downstream pathways, such as NF-κB, MAPK/ERK, and phosphatidylinositol 3-kinase (PI3K)/AKT pathway (Santarpia et al., 2010).

Wang L. et al. (2014) showed that miR-143 was able to act as a tumor inhibitor in glioma by targeting N-RAS. MiR-143 over-expression attenuated N-RAS expression, suppressed the PI3K/AKT and MAPK/ERK pathways, and reduced p65 accumulation in the nucleus of glioma cells. Moreover, miR-143 over-expression reduced tube formation from glioma endothelial cells, and inhibited invasion, migration, and angiogenesis and growth due to down-regulation of N-RAS both *in vivo* and *in vitro*. In addition, miR-143 sensitized the glioma cells to temozolomide that is the first-choice chemotherapy to treat glioma patients. Overall, they suggested that miR-143 might be a new treatment approach for glioma, as well as other RAS-driven malignancies (Wang L. et al., 2014).

The transmembrane heparan sulfate-bearing proteoglycan called syndecan-1 (SDC1) contributes to angiogenesis and invasion of the tumors by affecting MMP-9 (Oh et al., 2010; Ibrahim et al., 2012). When SDC1 is shed from the cell surface, it increased tumor growth and angiogenesis (Khotskaya et al., 2009). SDC1 over-expression revealed that MMP-9 transcription was activated by NF-κB. Additionally, proteolytically active MMP-9 has a role in the SDF-1 and heparanase-mediated shedding of SDC1 (Brule et al., 2006). Moreover, the molecules promoting the SDC1 shedding have been called “sheddases.” A soluble sheddase like MMP-7 and a cell surface sheddase like MMP-1 can mediate the shedding of SDC1 (Li et al., 2002), suggesting that SDC1 can be a substrate for more than one type of sheddase (Asuthkar et al., 2014). Asuthkar et al. (2014), reported that ionizing radiation (IR) could increase MMP-9, which in turn enhanced the SDC1 shedding, contributing to the tube-forming ability of medulloblastoma derived endothelial cells. Moreover, they demonstrated a new modulatory mechanism wherein MMP-9 caused miR-494 inhibition, leading to increased angiogenesis and SDC1 shedding. In addition, a luciferase reporter assay validated the 3′UTR of SDC1 as a direct target of miR-494. As result, they suggested that radiotherapy-induced angiogenesis could be partly caused by a MMP-9–miR-494–SDC1 feedback loop (Asuthkar et al., 2014).

Enhancer of zeste homolog 2 (EZH2) is a member of the poly-comb family of proteins, with a modulatory role in many cellular processes (Sparmann and van Lohuizen, 2006). It has been found that EZH2 is over-expressed in many cancers, such as GBM. Moreover, EZH2 was found to mediate angiogenesis and proliferation of GBM cells (Suvà et al., 2009; Smits et al., 2010). EZH2 is vital for transcriptional modulation of chromatin remodeling, nucleosome modification, and interacts with other transcription factors. EZH2 over-expression makes the G1 phase of the cell-cycle shorter and results in the cell accumulation in the S-phase (Bryant et al., 2007).

Sun et al. (2015) found that the expression level of miR-137 was down-regulated in GBM cells, and the low level of this miRNA was related to poor prognosis in GBM patients. MiR-137 ectopic expression suppressed angiogenesis and proliferation of GBM cells. Moreover, over-expression of miR-137 blocked GBM tumor growth *in vivo* and inhibited angiogenesis. Over-expression of EZH2, which is one of the direct targets of miR-137, suppressed the inhibitory effect of miR-137 on angiogenesis and proliferation. In addition, GBM patient tumor specimens revealed a converse correlation between the levels of EZH2 and miR-137. They suggested that miR-137 could be a biomarker in GBM patients, and might also be a new treatment approach for GBM treatment (Sun et al., 2015). Table 1 lists some angiogenesis-related miRNAs involved in GBM.

**TABLE 1:** Angiogenesis-related miRNAs responsible for GBM.

MiRNA	Loci	Expression status (up/down)	Targets	Models (*in vitro*, *in vivo*, human)	Kind of the cell-line	Reference
miR-125b	chr11: q24.1–q24.1	Down	VEGF	*In vitro*	Co-culture of HBMVECs, ACBRI-376, U87	Smits et al., 2012
miR-129-5p	chr7: q32.1–q32.1	Down	Wnt5a	Human, *in vitro*, *in vivo*	U251, LN229, A172, LN18, T98G	Zeng et al., 2018
miR-421	chrX: q13.2–q13.2	Down	MEF2D	Human, *in vitro*, *in vivo*	U251, U87	Liu et al., 2017
miR-1301-3p	chr2: 25551509–25551590	Down	N-Ras	Human, *in vitro*, *in vivo*	U87, U251, U118, LN229, A172, H4	Zhi et al., 2017b
miR-518b	chr19: q13.42–q13.42	Down	PDGFRB	Human, *in vitro*, *in vivo*	U87, U251	Xu X. et al., 2017
miR-107	chr10: q23.31–q23.31	Down	VEGF	*In vitro*, *in vivo*	U87, A172	Chen L. et al., 2016
miR-29c	chr1: q32.2–q32.2	Down	VEGF	Human, *in vitro*	U251, U87, T98G, A172, SHG44	Fan et al., 2013
miR-7-5p	chr9: q21.32–q21.32	Down	RAF1	Human, *in vitro*	HUV-EC-C, 293T	Liu et al., 2014
miR-566	chr3: 50210759–50210852	Up	VHL	*In vitro*, *in vivo*	U87	Xiao et al., 2016
miR-613	chr12: p13.1–p13.1	Down	VEGFA	Human, *in vitro*, *in vivo*	NHA, H4, U87, U251, SWO-38	Yu and Wang, 2017
miR-124-5p	chr8: p23.1–p23.1	Down	LAMB1	Human, *in vitro*, *in vivo*	U87, U251	Chen Q. et al., 2014
miR-124	chr8: p23.1–p23.1	Down	R-RAS, N-RAS	Human, *in vitro*, *in vivo*	U87, U251	Shi et al., 2014
miR-101	chr1: p31.3–p31.3	Down	EZH2	*In vitro*, *in vivo*	HBMVEC, C6, 293T, MCF-7, U118, U251, U373, U87	Smits et al., 2010
miR-143	chr5: q32–q32	Down	N-RAS	Human, *in vitro*, *in vivo*	U87, U251, HEK-293T	Wang L. et al., 2014
miR-558	chr2: p22.3–p22.3	Up	AGO2, eIF4E, HIF-2α	Human, *in vitro*, *in vivo*	NB-1643, SK-N-BE(2), NB-1691, IMR32, BE(2)-C, SK-N-AS, SH-SY5Y, SK-N-SH, HUVEC	Qu et al., 2016
miR-145	chr5: q32–q32	Down		*In vitro*, *in vivo*	HMEC, U87	Lu et al., 2015
miR-5096	Locus is not confident.	Up	Cx43	*In vitro*, *in vivo*	HMEC, U87	Thuringer et al., 2016
miR-137	chr1: p21.3–p21.3	Down	EZH2	Human, *in vitro*, *in vivo*	H4, U251, HUVEC, U87	Sun et al., 2015
miR-378	chr5: q32–q32	Up	VEGFR2	*In vitro*, *in vivo*	U87-miR-378, U87-GFP	Jamali et al., 2018
miR-520d-5p	chr19: 53720096–53720182 (+)	Down	PTTG1	Human, *in vitro*, *in vivo*	U87, U251, U118, LN229, A172, H4	Zhi et al., 2017a
miR-296	chr20: q13.32–q13.32	Up	PDGFR; VEGFR; HGS	*In vitro*, *in vivo*	HBMVEC, HUVEC, U87, 293T	Würdinger et al., 2008
miR-23b	chr9: q22.32–q22.32	Up	VHL	*In vitro*, *in vivo*	U87, LN229, U251, HUVEC	Chen L. et al., 2014
miR-16	chr13: 50048973–50049061 (−)	Down	VEGFR2/p38/NF−κB	Human, *in vitro*	HP75	Lu et al., 2018
miR-93	chr7: q22.1–q22.1	Up	VEGF, IL-8	Human, *in vitro*	U251, T98G	Fabbri et al., 2015
miR-137	chr1: p21.3–p21.3	Down	VEGF	*In vitro*	U87MG, LN18	Chakrabarti and Ray, 2015
miR-21	chr17: q23.1–q23.1	Up	SNALP and RhoB	*In vitro*, *in vivo*	GL261	Costa et al., 2015
miR-21	chr17: q23.1–q23.1	Up	HIF-1α, VEGF	Human, *in vitro*	A172, U87, T98G	Hermansen et al., 2016
miR−139−5p	chr11: q13.4–q13.4	Down	Flt1	Human, *in vitro*, *in vivo*	U87, SNB19, U251, LN308, LN229	Wang Q. et al., 2018
miR-494	chr14: q32.31–q32.31	Down	MMP-9, SDC1	Human, *in vitro*, *in vivo*	D283 Med, D425, D341,D458, H2402,H2405, UW228	Asuthkar et al., 2014
miR-218	chr4: p15.31–p15.31	Down	RTK	Human, *in vitro*, *in vivo*	U87MG, U87-SCR, U87-218	Mathew et al., 2014
miR-29b	chr7: q32.3–q32.3	Down	BCL2L2	*In vitro*	U251, U87, U373	Chung et al., 2015

## Long Non-Coding RNA Biogenesis

**FIGURE 3:**
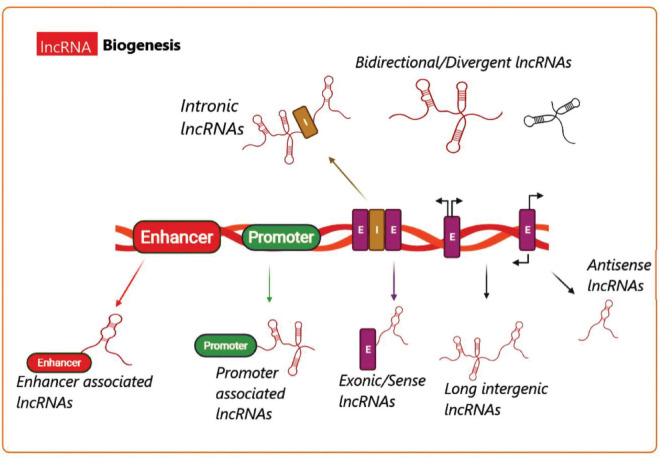
Long non-coding RNA biogenesis. A summary of the transcriptional origins of lncRNAs. These include entire or relatively natural anti-sense transcripts (NAT), coding genes between the genes, or into the introns, promoter (green), and enhancer (red) (E, exon; I, intron).

Long non-coding RNA (lncRNA) biogenesis has been proposed to be both cell-type and cell-stage specific, because it is controlled by the cell type as well as specific stimuli (Akerman et al., 2017). Transcription of various classes of lncRNAs from different DNA elements like intergenic regions, promoters, and enhancers within eukaryotic genomes is shown in Figure 3 (Wu et al., 2017). Different mechanisms are responsible for lncRNA biogenesis, including cleavage by ribonuclease P (RNaseP) for the generation of the mature ends, creation of snoRNA and protein (snoRNP) complexes as end-caps, as well as creation of the circular structures (Vicens and Westhof, 2014; Chen, 2016). In addition, certain subnuclear structures called “paraspeckles” have been found to surround particular lncRNAs in the course of their production (Naganuma and Hirose, 2013). Although the synthesis and regulation of all the known lncRNAs cannot be fully explained, nonetheless, additional investigations are underway to enhance our knowledge of the biogenesis as well as the function of many lncRNAs.

## LncRNAs and Angiogenesis in GBM

NPTX1 is a member of the pentraxin family produced as decamers or pentamers, and is able to bind to different biological ligands, including chromatin and bacteria (Boles et al., 2014). NPTX1 is involved in amyloid-beta-mediated neuronal death both in the mouse brain and in cultured cortical neurons (Qu and D’Mello, 2018). Furthermore, NFKB1 has been implicated in glioma development and progression (Qu and D’Mello, 2018).

Yu et al. (2020), showed that the lncRNA SLC26A4-AS1 had a role in glioma, with a new mechanism involving the transcription factors NPTX1 and NFKB1. They observed that the SLC26A4-AS1 level was lower in human glioma cells and tissues. Moreover, over-expression of either NPTX1 or SLC26A4-AS1 inhibited glioma cell aggressiveness and pro-angiogenic activity. SLC26A4-AS1 up-regulated NPTX1 through recruitment of NFKB1 in the NPTX1 promoter. Additionally, silencing of NFKB1 or NPTX1 resulted in restoring the pro-angiogenic and aggressive features of the glioma cells, which had been inhibited by SLC26A4-AS1 expression. Thus, SLC26A4-AS1 enhanced the transcriptional activity of NPTX1 *via* recruiting NFKB1 and hence had an anti-angiogenic effect on the glioma cells (Yu et al., 2020).

It has been reported that lncRNA PAXIP1-AS1 promotes cell death, so that its silencing can contribute to cell survival (Weirick et al., 2018). Aberrant expression of KIF14 (kinesin-like protein) has been shown in glioma, which is linked with increased aggressiveness (Wang Q. et al., 2013). The inhibition of ETS1 (avian erythroblastosis virus E26 oncogene homolog-1), a protooncogene transcription factor with a role in glioma development, may be a possible strategy for glioma treatment (Sahin et al., 2005).

Xu H. et al. (2019) found that the expression level of lncRNA PAXIP1-AS1 was increased in glioma cells and tissues, and was correlated with up-regulated KIF14. LncRNA PAXIP1-AS1 is located in the glioma cell nucleus, where it could increase the promoter activity of KIF14 through recruitment of the transcription factor ETS1. In addition, over-expression of LncRNA PAXIP1-AS1 increased angiogenesis, invasion, and migration of HUVECs co-cultured with glioma cells *in vitro*, and also increased the growth rate of glioma xenograft tumors in nude mice. The lncRNA PAXIP1-AS1/ETS1/KIF14 axis could be considered as a target for glioma therapy, because of its contribution to invasion, migration, and angiogenesis in glioma tumors (Xu H. et al., 2019).

Hypoxia-inducible factors (HIFs) govern the physiological adaptation of tissue to varying levels of oxygenation, acting as crucial transcriptional regulators at organism, tissue, and cellular levels (Palazon et al., 2017). HIF-1α has been the most broadly investigated of the HIFs, with a key role in driving angiogenesis (Liu et al., 2019). Recently, a study by Liu et al. (2019) investigated the relationship between H19 and the miR-138/HIF-1α/VEGF axis, through which lncRNA H19 may influence angiogenesis in glioma. They found that H19 knock-down suppressed angiogenesis, proliferation, and migration of glioma cells. According to the results, miR-138 was found to be a target of H19. Because HIF-1α was in turn a target for miR-138, this finding explained why H19 affected angiogenesis, migration as well as proliferation of the glioma cells *through* targeting HIF-1α and affecting VEGF expression. The up-regulation of lncRNA H19 in glioma cells, suggested it could function as one of the ceRNAs for miR-138, and thus promote angiogenesis, invasion, migration, and proliferation by increasing HIF-1α expression (Liu et al., 2019).

Microchromosome maintenance protein 3 (MCM3) is a key factor involved in DNA replication. Furthermore, MCM3AP is an acetyltransferase that reacts with MCM3 to affect the chromatin structure. However, over-expression of MCM3AP inhibited DNA replication by causing cell cycle arrest in the S phase, and inhibited proliferation in a MCM3AP acetylase dependent manner (Poole et al., 2012). The location of the MCM3AP gene has been reported to be on human chromosome 21, and its expression is altered in various cancer types (Kuwahara et al., 2016). Yang et al. (2018) reported that the lncRNA called MCM3AP antisense 1 (MCM3AP-AS1) is up-regulated in glioma-derived endothelial cells (GECs), and led to the down-regulation of miR-211. MCM3AP-AS1 knock-down inhibited the number, migration, and viability of the GECs, and could suppress GBM angiogenesis *in vitro* as well increasing miR-211 expression. A luciferase reporter assay showed tate miR-211 targeted KLF5 3′-UTR to suppress the expression of KLF5. Additionally, they showed that knock-down of MCM3AP-AS1 decreased AGGF1 and KLF5 expression through up-regulating miR-211. KLF5 knock-down reduced the expression of AGGF1 *via* transcriptional repression, and also suppressed ERK1/2 activation and PI3K/AKT signaling pathways. The MCM3AP-AS1/miR-211/KLF5/AGGF1 axis contributes to the modulation of GBM angiogenesis, and thus may be a promising target for anti-angiogenesis treatment of glioma (Yang et al., 2018).

According to another study, EGF-like domain 7 (EGFL7) is an endothelial cell secreted factor involved in the formation of vascular tubes (Parker et al., 2004). Moreover, NFAT5 is a member of the family of nuclear factor of activated T-cells (NFAT) transcription factors. NFAT5 also has a role in regulating tumor biology (Parker et al., 2004), and can enhance the expression of pro-angiogenic factors (Amara et al., 2016). Yu et al. (2017), showed the transcription factor NFAT5 and the lncRNA SBF2 antisense RNA 1 (SBF2-AS1) were both remarkably increased in GBM cell-lines and glioma tissue samples. Knock-down of BF2-AS1 and NFAT5 led to suppression of GBM-mediated angiogenesis. In addition, down-regulation of NFAT5 suppressed the expression of SBF2-AS1 at a transcriptional level. SBF2-AS1 knock-down suppressed GBM cell-driven angiogenesis by promoting the inhibitory effects of miR-338-3p on EGFL7 expression. Therefore, the NFAT5/SBF2-AS1/miR 338-3p/EGFL7 pathway may be a new target for the anti-angiogenic treatment of glioma (Yu et al., 2017). Table 2 lists some angiogenesis-related lncRNAs involved in GBM.

**TABLE 2:** Various angiogenesis-related lncRNAs in GBM.

LncRNAs	Loci	Expression status	Targets	Model (*in vitro*, *in vivo*, human)	Kind of cells	Reference
SLC26A4-AS1	chr7: q22.3–q22.3	Down	NPTX1	Humans, *in vitro*, *In vivo*	T98G, HS683, U251	Yu et al., 2020
PAXIP1-AS1	chr7: q36.2–q36.2	Up	ETS1	Human, *in vitro*, *in vivo*	TJ905, HS 683, H4, SHG-44, Has	Xu H. et al., 2019
SNHG15	chr7: p13–p13	Up	miR-153	Human, *in vitro*, *in vivo*	hCMECs	Ma et al., 2017
TUG1	chr22: q12.2–q12.2	Up	miR-299 (VEGFA)	Human, *in vitro*, *in vivo*	U251 MG, U87MG, HEK293T	Cai et al., 2017
lncRNA-LINC01116	–	Up	VEGFA	Human, *in vitro*, *in vivo*	U251, A172, U87MG, U118MG, HUVEC, HEK293T	Ye et al., 2020
lncRNA H19	chr11: p15.5–p15.5	Up	miR-29a	Human, *in vitro*, *in vivo*	HBMVEC	Jia et al., 2016
lncRNA H19	chr11: p15.5–p15.5	Up	miR-138/HIF-1α/VEGF138/HIF-1α	*In vitro*	U87, A172, U373, HEB	Liu et al., 2019
MCM3AP-AS1	chr21: q22.3–q22.3	Up	miR-211 (KLF5 and AGGF1)	*In vitro*, *in vivo*	hCMEC/D3, U87, HEK293T	Yang et al., 2018
SBF2-AS1	chr11: p15.1	Up	miR-338-3p, EGFL7	Human, *in vitro*, *in vivo*	U87, U118, HEK293T	Yu et al., 2017

## Circular RNA Biogenesis

**FIGURE 4:**
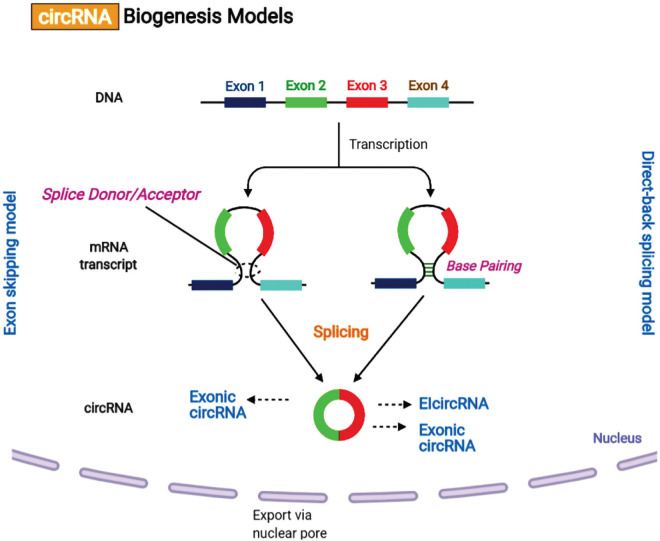
Circular RNA biogenesis mechanisms. (A) Lariat-driven circularization or exon skipping model. The splice donor at the 3′ end of Exon 1 is covalently attached to the splice acceptor at the 5′ end of Exon 4 creating a lariat *via* exon skipping. Then, the spliceosome removes the introns for creating a circular RNA. (B) Intron pairing-driven circularization or direct-back splicing model. The successive introns form a circular structure by base pairing, and then are eliminated for forming an exonic circRNA or re-attached to form an EIcircRNA.

Recently, it has been reported that the biogenesis of circular RNAs (circRNAs) *via* the back-splicing mechanism differs from the canonical mechanism involving linear RNA splicing. circRNAs are categorized into intronic-circRNAs, exon-intron circRNAs, and exonic-circRNAs. Jeck et al. (2013) suggested two models for circRNAs formation, intron-pairing-driven circularization and lariat-driven circularization (Figure 4). It was shown that exon circularization is based on flanking intronic complementary sequences. The alternation of inverted repeated Alu pairs will result in alternative circularization, leading to multiple circRNA transcripts being generated from a single gene. Besides, RNA-binding proteins (RBPs) may function as inhibitors or activators of circRNA processing. It has been found that over one-third of circRNAs biosynthesis is dynamically regulated by the alternative splicing factor called Quaking (QKI).

Moreover, QKI motifs are sufficient to initiate the *de novo* synthesis of circRNA from linear normally spliced transcripts. However, ADAR1, a double-strand RNA-editing enzyme, can antagonize circRNA expression by disrupting the stem structure. The precise mechanism of circRNAs biosynthesis remains to be fully elucidated.

## Circular RNAs and Angiogenesis in GBM

Forkhead box (FOX) proteins are one of the evolutionarily conserved transcription factor families, with a DNA-binding domain, which have many important roles in the regulation of cell functions (Potente et al., 2005). The protein known as “angiogenic factor with G-patch and FHA domains 1” (AGGF1) has been recognized as a novel pro-angiogenic protein, which is highly expressed in vascular endothelial cells, and contributes to angiogenesis in different cancers (Tian et al., 2004). Recently, He et al. (2018) investigated the molecular mechanism and potential role of circ-SHKBP1 in modulating angiogenesis in U87 glioma endothelial cells (GECs). Circ-SHKBP1 expression was considerably up-regulated in the GECs in comparison to astrocyte-exposed endothelial cells (AECs). They found that knock-down of the circ-SHKBP1 suppressed the tube formation, migration, and viability of GECs. Also, they observed that miR-544a/miR-379 was functionally targeted by circ-SHKBP1 and knock-down of circ-SHKBP1 caused the decreased expression of miR-379/miR-544a in the GECs. FOXP1/2 were found to be targets of miR-544a/miR-379. In addition, FOXP1/FOXP2 silencing suppressed the tube formation, migration, and viability of GECs. The result of this study demonstrated that FOXP1/FOXP2 promoted AGGF1 expression at the transcriptional level and knock-down of AGGF1 inhibited tube formation, migration, and viability of GECs by the PI3K/AKT and ERK1/2 pathways. Overall, circ-SHKBP1 modulated GEC angiogenesis *via* miR-379/FOXP2 and miR-544a/FOXP1 pathways, and these results could be used in a combination therapy for glioma (He et al., 2018).

The splicing factor Serine and Arginine Rich Splicing Factor 1 (SRSF1) is implicated in several biomolecular processes, and is up-regulated in several kinds of cancers (Martínez-Terroba et al., 2018). Moreover, a study by Barbagallo et al. (2019) showed a physical interaction between circSMARCA5 and SRFS1. They examined the expression level of the two mRNA isoforms of VEGFA, the anti-angiogenic (Iso8b) and the pro-angiogenic (Iso8a), and found the ratio was affected by SRSF1. They analyzed 31 GBM patient biopsies and 20 unaffected brain parenchyma specimens (UC) (Barbagallo et al., 2019). The ratio of Iso8a to Iso8b was higher in GBM biopsies compared to UC, and was negatively correlated with circSMARCA5 expression. The ratio was lower in U87-MG cells which over-expressed circSMARCA5 compared to control cells. Moreover, the vascular microvessel density in the GBM specimens showed a negative correlation with circSMARCA5 expression, which had a positive correlation with SRSF1 levels, and with the ratio of Iso8a to Iso8b. According to Kaplan–Meier survival analysis, the GBM patients with a lower expression of circSMARCA5 had a shorter progression free and overall survival rate than those with higher expression of circSMARCA5. They proposed that circSMARCA5 could affect the ratio of the pro- to anti-angiogenic VEGFA isoforms in GBM, and could be a prognostic bio-marker for anti-angiogenesis treatment (Barbagallo et al., 2019). Table 3 lists various angiogenesis-related circRNAs reported to be involved in GBM.

**TABLE 3:** Various angiogenesis-related circular RNAs involved in GBM.

Circular RNAs	Loci	Expression status	Target	Models (*in vitro*, *in vivo*, human)	Type of cell-line	Reference
circ-SHKBP1	chr19: q13.2–q13.2	Up	miR544a/FOXP1, miR-379/FOXP2	Human, *in vitro*, *in vivo*	hCMEC/D3, U87MG	He et al., 2018
circSMARCA5	chr4: q31.1–q31.21	Down	SRSF1	Human, *in vitro*	A172, CAS-1, U87-MG	Barbagallo et al., 2019
circ_0010729	–	Up	miR-186/HIF-1a	*In vitro*	HUVECs, HEK293T	Dang et al., 2017
cZNF292	chr6: 87920168–87928449 (+)	Up	p-VEGFR-1/2, VEGFR-1/2, EGFR	*In vitro*	U251, U87MG	Yang et al., 2016

## Biogenesis of Exosomes

**FIGURE 5:**
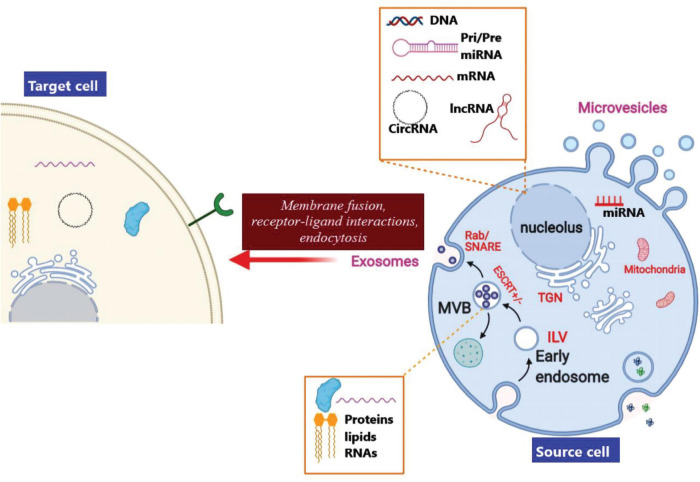
Biogenesis, cargo loading, and secretion of exosomes. Invagination of the endocytic membrane leads to the creation of ILVs and finally to secretion of exosomes. In the course of the maturation process, cargo molecules (proteins, lipids, and RNAs) are loaded into ILVs *via* an ESCRT–dependent pathway or an ESCRT–independent pathway. Maturation of the initial endosomes results in the formation of MVBs that are transferred to the trans-Golgi network (TGN) in order to recycle the endosomes. The MVBs can also be delivered to the lysosomes for degradation, or moved along micro-tubules for fusion with the plasma membrane in order to release the exosomes into the extra-cellular space. Moreover, MVB fusion with the plasma membrane involves the SNARE and Rab GTPase complexes. Finally, exosomal contents originating from the source cells may be taken up by the target cells *via* a direct membrane fusion, receptor-ligand interactions, or by endocytosis,

Exosomes are membrane-encapsulated vesicles resulting from the interior budding of the plasma membrane to first form endosomes, and the membrane partially invaginates and forms buds containing the cytoplasmic contents to form intraluminal vesicles (ILVs) (McAndrews and Kalluri, 2019). The late endosomal structures which consist of dozens of ILVs are called multi-vesicular bodies (MVBs), that may be exported to the trans-Golgi network (TGN) to recycle the endosomes, delivered to the lysosomes for degrading the contents, or fused with the plasma membrane and released into the extra-cellular space as exosomes (Williams and Urbé, 2007; Figure 5). The endosomal sorting complex required for transport (ESCRT) is responsible for biogenesis as well as secretion of the exosomes (Kalluri and LeBleu, 2020). ESCRT contains four components, ESCRT−0, ESCRT-I, ESCRT-III, and ESCRT-II together with the respective proteins Tsg101 ALIX, and VPS4. ESCRT-0 organizes the ubiquitinated cargo proteins in a lipid domain, while ESCRT-I and ESCRT-II trigger membrane deformation to form a stable membrane neck. Finally, the Vps4 complex is recruited to ESCRT−III in order to cut off the neck, release the exosomes, and then recycle the ESCRT−III complex (Juan and Fürthauer, 2018).

## Exosomal Non-Coding RNAs and Angiogenesis

Kruppel-like factor (KLF)-2 and KLF4 belong to the KLF sub-family of zinc finger proteins (Li et al., 2016). These KLF proteins act as a tumor inhibitor in several cancer types, and their ectopic expression may suppress the proliferation of the cells (Li et al., 2016). KLF2 inhibits angiogenesis by suppressing VEGFR2 promoter activity (Bhattacharya et al., 2005), while KLF4 maintains the endothelial barrier integrity *via* increasing the promoter activity of the tight junction–associated proteins like occludin, ZO-1, and claudin-5 (Ma et al., 2014).

In one study, GBM cells were found to generate a large number of exosomes. The content of miR-182-5p in the exosomes from the GBM cells under hypoxic conditions was higher, compared to parental cells under normoxic conditions. Moreover, exosomal miR-182 5p inhibited its targets KLF2 and KLF4, resulting in higher VEGFR production, thereby increasing tumor angiogenesis (Li et al., 2020). Additionally, the exosomal miR-182-5p also decreased tight junction-related proteins (occludin, ZO-1, and claudin-5), hence, increasing trans endothelial tumor cell migration and vascular permeability. MiR-182-5p knock-down decreased tumor proliferation and angiogenesis. It has been reported that the expression level of miR-182-5p in cerebrospinal fluid samples and serum from GBM patients was increased and its expression levels were inversely correlated with patient prognosis (Li et al., 2020).

The ERBB receptor feedback inhibitor 1 (ERRFI1) is a member of the scaffolding adapter protein family, with a contribution to the EGFR signaling pathway (Ahn et al., 2012). The cells that expressed higher levels of EGFR had correspondingly lower levels of ERRF1 (Cairns et al., 2018). The EGFR/mitogen activated protein kinase (MAPK) pathway is known to contribute to glioma progression (Qu et al., 2012). Recently, Wang et al. (2020) evaluated the regulatory role of exosomal miR-148a-3p in glioma. In this research, the exosomes were isolated from glioma cells and healthy human astrocytes, and the expression level of exosomal miR-148a-3p was measured by RT-qPCR. A dual luciferase reporter assay was used to confirm the direct binding of ERRFI1 to miR-148a-3p. They found that miR-148a-3p was strongly expressed whereas ERRFI1 showed low expression in glioma cells. Moreover, miR-148a-3p was plentiful in the glioma cell-derived exosomes, and could be transported to the HUVECs in culture to promote angiogenesis and proliferation. Furthermore, inhibition of ERRFI1 activated the EGFR/MAPK pathway *via* miR-148a-3p. This mechanism was suggested to be responsible for the promotion of angiogenesis and tumorigenesis by exosomal miR-148a-3p (Wang et al., 2020).

The phosphatase and tensin homolog deleted on chromosome 10 (PTEN) is a tumor inhibitor gene, that is inactivated in several cancers (Song et al., 2012). PTEN modulates different cellular processes like survival, proliferation, energy metabolism, and cellular architecture *via* activation of the PI3K-AKT-mTOR pathway (Song et al., 2012). Recently Wang and co-workers (Wang et al., 2019) conducted a study to investigate a novel RNA-based treatment for glioma. They assessed whether exosomes derived from glioma stem cells (GSCs), which contained miR-26a, could affect microvascular endothelial cells and angiogenesis in glioma. The results of this study showed that the expression of miR-26a was up-regulated and PTEN was down-regulated in the glioma cells. Moreover, miR-26a could activate the PI3K/Akt pathway by targeting PTEN. They observed that miR-26a increased angiogenesis, tube formation, migration, and proliferation of HBMECs *in vitro*. GSC-derived exosomes which over-expressed miR-26a caused an increase in HBMEC angiogenesis and proliferation *in vitro* by PTEN suppression. The pro-angiogenic effect of the GSC-derived exosomes containing miR-26a *in vivo* confirmed the *in vitro* results. They concluded that GSC-derived exosomal miR-26a contributed to HBMEC angiogenesis (Wang et al., 2019).

OCT4 and MYC are two of the canonical stem cell factors responsible for progression of a wide range of solid tumors, particularly glioma. Notably, MYC can bind to the EGFR/EGFRvIII promoter region to increase the malignant phenotype of glioma cells (Zhao et al., 2017). OCT4 has also been found to increase the likelihood of recurrence of glioma (Wu et al., 2016). Furthermore, OCT4 and MYC are also involved in the formation of neovasculature. MYC can bind to the angiopoietin-like 4 (Angptl4) promoter region to trigger its expression, and increase angiogenesis (Katanasaka et al., 2013). Abnormal expression of OCT4 in HUVECs transformed them into endothelial progenitor cells, and increased their angiogenic potential (Mou et al., 2016). In addition, OCT4 and MYC have been associated with the function and expression of several miRNAs (Cruz-Santos et al., 2016).

Chen X. et al. (2019), conducted a study to examine the contribution of miR-9 to glioma pathogenesis. They observed that miR-9 containing exosomes were released from glioma cells, and were taken up by vascular endothelial cells, resulting in increased angiogenesis. The results showed that PHD3, PTCH1, THBS2, and COL18A1 were the direct targets of miR-9, serving to increase the malignant phenotype in the glioma cells. OCT4 and MYC could bind to the promoter region of miR-9 to stimulate its transcription. Their findings demonstrated that miR-9 was involved in the pathogenesis of glioma, and therefore may be a promising target for treating glioma (Chen X. et al., 2019).

The antisense transcript of hypoxia-inducible factor (AHIF) contributes to tumor progression (Tasharrofi et al., 2016; Zhang et al., 2016). A study by Dai et al. (2019) showed the remarkable up-regulation of AHIF in radioresistant GBM cells and in GBM tissues. In response to radiation, AHIF expression was further up-regulated. AHIF knock-down in GBM cells reduced the viability and invasion, and enhanced the proportion of apoptotic cells. AHIF over-expression in GBM cells had the opposite effect by increasing viability and invasion, and reducing apoptosis. Moreover, the exosomes extracted from the AHIF-knockdown GBM cells suppressed radioresistance, invasion, and viability, while the exosomes from AHIF-over-expressing GBM cells increased radioresistance, invasion, and viability. This study revealed that AHIF secreted in exosomes promoted GBM development and radioresistance, suggesting AHIF could be a treatment target in GBM (Dai et al., 2019). Table 4 lists some angiogenesis-related exosomal ncRNAs reported to be involved in GBM.

**TABLE 4:** Various angiogenesis-related exosomal non-coding RNAs in GBM.

Cargo	Loci	Expression status	Targets	Model (*in vitro*, *in vivo*, humans)	Kind of cells	Reference
linc-POU3F3	chr2: q12.1–q12.1	Up	Endothelial cells	Human, *in vitro*	A172, U251, U87-MG, 293T, T98G, HBMECs	Lang H. et al., 2017
lncRNA HIF1A	chr12: q24.31–q24.31	Up	HIF−1α	Human, *in vitro*	U251-MG, U87-MG, T98G, A172	Dai et al., 2019
lincRNA-CCAT2	chr 8: q24.21	Up	VEGFA, TGFβ	Human, *in vitro*	A172, U251, U87-MG, T98G, 293T, HUVECs	Lang H.-L. et al., 2017
miR-9	chr1: q22–q22	Up	COL18A1, THBS2, PTCH1, PHD3	Human, *in vitro*, *in vivo*	A172, U251, U87, BT325, SHG44	Chen X. et al., 2019
miR-26a	chr3: p22.2–p22.2	Up	PTEN	Human, *in vitro*	SHG-44, BT325, T98G, A172, U251, HEB, HBMECs	Wang et al., 2019
miR-182-5p	chr7: q32.2–q32.2	Up	KLF4, KLF2	Human, *in vitro*, *in vivo*	HUVEC, U-251MG, H4, A-172, U-118MG, LN-18, U-87MG	Li et al., 2020
miR-148a-3p	chr7: p15.2–p15.2	Up	ERRFI1	Human, *in vitro*, *in vivo*	U-138-MG, U251-MG, LN229, HA, HUVECs	Wang et al., 2020
miR-21	chr17: q23.1–q23.1	Up	VEGF/VEGFR2	*In vitro*	U-251, hbECs	Sun et al., 2017

## Conclusion

As tumors grow and develop from a small size they must undergo an angiogenic switch leading to the release of signaling molecules needed for the creation of new capillary sprouts from the existing blood vessels. Moreover, a complex interaction drives tumor angiogenesis, forming a balance between anti-angiogenic factors (TSP-1/TSP-2) and pro-angiogenic factors (PDGF/PDGFR, VEGF/VEGFR) within the tumor microenvironment. Moreover, the tissue degradation and remodeling by MMPs is governed by tissue inhibitors of metalloproteinases (TIMPs), which also affect angiogenesis in tumors. In addition, tumor oncogenes or tumor suppressors control cell migration and viability, and may be affected by hypoxia (MYC and HIF) with a role in GBM angiogenesis. NcRNAs, including miRNAs have been found to control gene expression in a post-transcriptional manner. MiRNAs can modulate many important normal physiological processes, including differentiation, apoptosis, and proliferation, as well as pathological processes such as oncogenesis and angiogenesis. Accumulating evidence suggests that ncRNAs can directly regulate angiogenesis *via* targeting crucial angiogenic factors and signaling pathways in GBM. Discovering the underlying mechanisms behind the angiogenesis regulation by ncRNAs is of high importance because it might lead to the advances in novel treatments for GBM patients. Many ncRNAs that can play a role in angiogenesis, including lncRNAs have been shown to exist within the human genome. Nonetheless, the exact mechanisms of many lncRNAs are still unknown. One intriguing way that ncRNAs can affect the angiogenesis process is by being secreted inside exosomes from GBM cells, and then taken up by neighboring endothelial cells where they can affect gene expression of pro-angiogenic factors. Exosomes may also be employed as a delivery route for exogenous RNA-based therapeutics intended to combat GBM angiogenesis.

## Author Contributions

HM, NR, and MRH contributed to conception, design, statistical analysis, and drafting of the manuscript. EB, KM, YM, MHP, AR, ZR, and AM contributed to data collection and manuscript drafting. All authors approved the final version for submission.

## Conflict of Interest

The authors declare that the research was conducted in the absence of any commercial or financial relationships that could be construed as a potential conflict of interest.

## Publisher’s Note

All claims expressed in this article are solely those of the authors and do not necessarily represent those of their affiliated organizations, or those of the publisher, the editors and the reviewers. Any product that may be evaluated in this article, or claim that may be made by its manufacturer, is not guaranteed or endorsed by the publisher.
